# 2-[(8-Meth­oxy­carbonyl-4b,8-dimethyl-4b,5,6,7,8,8a,9,10-octa­hydro­phenan­thren-3-yl)amino]-3,5-dinitro­benzoic acid ethyl acetate monosolvate

**DOI:** 10.1107/S1600536812032278

**Published:** 2012-08-11

**Authors:** Bihai Tong, Ye Zhang

**Affiliations:** a College of Metallurgy and Resources, Anhui University of Technology, Maanshan 243002, People’s Republic of China; b Department of Chemistry and Engineering Technology, Guilin Normal College, Guilin 541001, People’s Republic of China

## Abstract

The title compound, C_25_H_27_N_3_O_8_·C_4_H_8_O_2_, has a diterpene skeleton in which the fused cyclo­hexane rings exhibit chair and half-chair conformations. An intra­molecular C—H⋯O hydrogen bond occurs. In the crystal, N—H⋯O, O—H⋯O and C—H⋯O hydrogen bonds are observed.

## Related literature

For the synthesis of *cis*-deisopropyl­dehydro­abietate derivatives, see: Fonseca *et al.* (2001 ▶); Baleizao *et al.* (2004 ▶); Feio *et al.* (1999 ▶). For a related structures, see: Wang *et al.* (2006 ▶); Hamodrakas *et al.* (1978 ▶). For uses of dehydro­abietic acid (DAA), see: Bhatnagar (1983 ▶, 1984 ▶). For the geometry of diterpenic compounds, see: Allen *et al.* (1991 ▶);
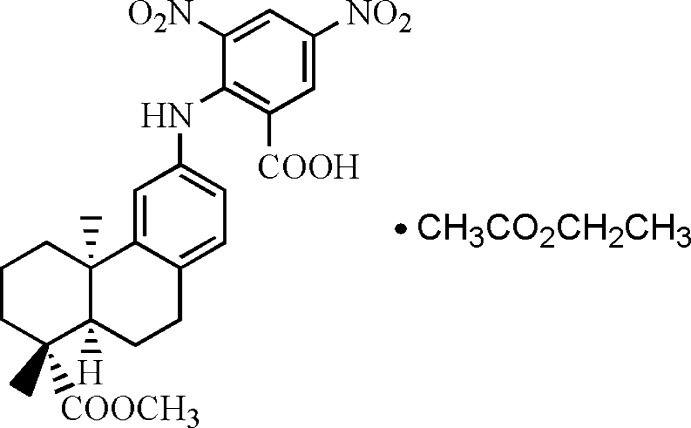


## Experimental

### 

#### Crystal data

C_25_H_27_N_3_O_8_·C_4_H_8_O_2_
*M* *_r_* = 585.60
Monoclinic, 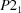

*a* = 7.649 (4) Å
*b* = 13.591 (8) Å
*c* = 14.399 (8) Å
β = 101.371 (7)°
*V* = 1467.5 (15) Å^3^
*Z* = 2
Mo *K*α radiation
μ = 0.10 mm^−1^
*T* = 296 K
0.47 × 0.38 × 0.12 mm

#### Data collection

Bruker SMART CCD area-detector diffractometer
Absorption correction: multi-scan (*SADABS*; Bruker, 2002 ▶) *T*_min_ = 0.954, *T*_max_ = 0.988
9128 measured reflections
5885 independent reflections
4688 reflections with *I* > 2σ(*I*)
*R*_int_ = 0.016

#### Refinement

*R*[*F* ^2^ > 2σ(*F* ^2^)] = 0.041
*wR*(*F* ^2^) = 0.107
*S* = 1.03
5885 reflections
385 parameters
1 restraint
H-atom parameters constrained
Δρ_max_ = 0.13 e Å^−3^
Δρ_min_ = −0.17 e Å^−3^

### 

Data collection: *SMART* (Bruker, 2002 ▶); cell refinement: *SAINT* (Bruker, 2002 ▶); data reduction: *SAINT*; program(s) used to solve structure: *SHELXS97* (Sheldrick, 2008 ▶); program(s) used to refine structure: *SHELXL97* (Sheldrick, 2008 ▶); molecular graphics: *SHELXTL* (Sheldrick, 2008 ▶); software used to prepare material for publication: *SHELXL97*.

## Supplementary Material

Crystal structure: contains datablock(s) I, global. DOI: 10.1107/S1600536812032278/kp2429sup1.cif

Structure factors: contains datablock(s) I. DOI: 10.1107/S1600536812032278/kp2429Isup2.hkl

Supplementary material file. DOI: 10.1107/S1600536812032278/kp2429Isup3.cml

Additional supplementary materials:  crystallographic information; 3D view; checkCIF report

## Figures and Tables

**Table 1:** Hydrogen-bond geometry (Å, °)

*D*—H⋯*A*	*D*—H	H⋯*A*	*D*⋯*A*	*D*—H⋯*A*
N1—H1*S*⋯O6	0.86	2.02	2.654 (3)	129
O5—H5⋯O2*S* ^i^	0.82	1.87	2.676 (3)	168
C4*S*—H4*S*3⋯O2	0.96	2.54	3.332 (4)	139
C15—H15*B*⋯O8	0.97	2.53	3.326 (4)	140

Symmetry code: (i) 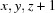.

**Fig. 1.**
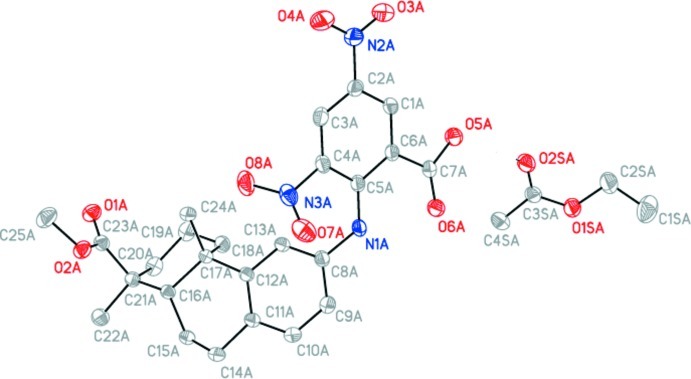
The molecular structure of (I), with atom labels and 25% probability displacement ellipsoids for non-H atoms.

**Fig. 2.**
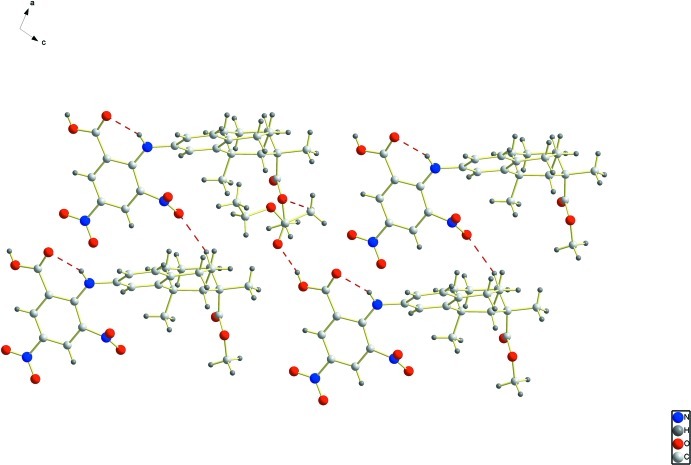
A packing diagram of the title compound, showing hydrogen bonds drawn as dashed lines.

## supplementary crystallographic information

### Comment

Dehydroabietic acid (DAA) is one of the isomerides in the renewable rosin. It is widely used in the fields such as paint, adhesives, printing ink (Bhatnagar, 1983), papermaking, and rubber food (Bhatnagar, 1983). Like some natural drug, it has an aromatic diterpene structure with three rings. It also has three sterogenic centres but their absolute configuration cannot be determined by this analysis. The compound comprises a reactive carboxy group and DAA molecule might be modified to obtain some multifunctional derivatives which can be used as high added value products like novel fluorescence derivatization reagents and efficient but low toxic medicines through constructing aromatic or heteroaromatic ring on DAA' s skeleton. Methyl *cis*-deisopropyldehydroabietate can be easily synthesized from DAA (Fonseca *et al.*, 2001). It provides a convenient starting material for the construction of other derivatives. Some of these derivatives lacking the isopropyl group are also antimicrobial agents (Feio *et al.*, 1999). The molecular structure of the title compound (I) (Fig. 1), as typical of diterpenic compounds (Allen *et al.*, 1991), shows a *trans* junction of rings A (defined by C16, C17, C18, C19, C20, C21) and B (defined by C11, C12, C17, C16, C15, C14) with two methyl groups in axial positions of the six membered rings. The torsion angles show a chair and a half-chair conformation for rings A and B, respectively. The overall geometry of (I) is comparable to that found for methyl dehydroabietate (Hamodrakas *et al.*, 1978), apart from the substituted 2,4,6 - trinitrophenylamino and methylgroups at the benzene ring.

### Experimental

The title compound was obtained by refluxing methyl 13-amino- *cis*-deisopropyldehydroabietate and 2-chloro-3, 5- dinitrobenzoic acid in ethanol in the presence of copper powder and potassium carbonate to give the title compound as a yellow precipitate in 87.7% yield. Recrystallization from ethyl acetate gave orange block-like crystals suitable for an X-ray diffraction experiment. Anal.Calcd. for C_29_H_35_N_3_O_10_: C, 59.48, H, 6.02, N, 7.18%. Found:*C*, 58.90, H, 6.56, N, 7.00%.

### Refinement

H atoms were positioned geometrically and refined using a riding model (including free rotation about the ethanol C—C bond), with C—H = 0.95–0.99 Å and with *U*_iso_(H) = 1.2 (1.5 for methyl groups) *U*_eq_(C).

### Crystal data

**Table:**

C_25_H_27_N_3_O_8_·C_4_H_8_O_2_	*F*(000) = 620
*M**_r_* = 585.60	*D*_x_ = 1.325 Mg m^−^^3^
Monoclinic, *P*2_1_	Mo *K*α radiation, λ = 0.71073 Å
Hall symbol: P 2yb	Cell parameters from 3055 reflections
*a* = 7.649 (4) Å	θ = 2.8–24.0°
*b* = 13.591 (8) Å	µ = 0.10 mm^−^^1^
*c* = 14.399 (8) Å	*T* = 296 K
β = 101.371 (7)°	Block, orange
*V* = 1467.5 (15) Å^3^	0.47 × 0.38 × 0.12 mm
*Z* = 2	

### Data collection

**Table:**

Bruker SMART CCD area-detector diffractometer	5885 independent reflections
Radiation source: fine-focus sealed tube	4688 reflections with *I* > 2σ(*I*)
Graphite monochromator	*R*_int_ = 0.016
ω scans	θ_max_ = 27.5°, θ_min_ = 2.8°
Absorption correction: multi-scan (*SADABS*; Bruker, 2002)	*h* = −9→9
*T*_min_ = 0.954, *T*_max_ = 0.988	*k* = −17→14
9128 measured reflections	*l* = −16→18

### Refinement

**Table:**

Refinement on *F*^2^	Primary atom site location: structure-invariant direct methods
Least-squares matrix: full	Secondary atom site location: difference Fourier map
*R*[*F*^2^ > 2σ(*F*^2^)] = 0.041	Hydrogen site location: inferred from neighbouring sites
*wR*(*F*^2^) = 0.107	H-atom parameters constrained
*S* = 1.03	*w* = 1/[σ^2^(*F*_o_^2^) + (0.058*P*)^2^ + 0.0376*P*] where *P* = (*F*_o_^2^ + 2*F*_c_^2^)/3
5885 reflections	(Δ/σ)_max_ = 0.024
385 parameters	Δρ_max_ = 0.13 e Å^−^^3^
1 restraint	Δρ_min_ = −0.17 e Å^−^^3^

### Special details

**Table:**

Geometry. All e.s.d.'s (except the e.s.d. in the dihedral angle between two l.s. planes) are estimated using the full covariance matrix. The cell e.s.d.'s are taken into account individually in the estimation of e.s.d.'s in distances, angles and torsion angles; correlations between e.s.d.'s in cell parameters are only used when they are defined by crystal symmetry. An approximate (isotropic) treatment of cell e.s.d.'s is used for estimating e.s.d.'s involving l.s. planes.
Refinement. Refinement of *F*^2^ against ALL reflections. The weighted *R*-factor *wR* and goodness of fit *S* are based on *F*^2^, conventional *R*-factors *R* are based on *F*, with *F* set to zero for negative *F*^2^. The threshold expression of *F*^2^ > σ(*F*^2^) is used only for calculating *R*-factors(gt) *etc*. and is not relevant to the choice of reflections for refinement. *R*-factors based on *F*^2^ are statistically about twice as large as those based on *F*, and *R*- factors based on ALL data will be even larger.

### Fractional atomic coordinates and isotropic or equivalent isotropic displacement parameters (Å^2^)

**Table:**

	*x*	*y*	*z*	*U*_iso_*/*U*_eq_	
N1	0.4541 (2)	0.20663 (15)	1.11523 (11)	0.0506 (4)	
H1S	0.4165	0.2369	1.1599	0.061*	
N2	1.1161 (3)	0.01668 (16)	1.21945 (16)	0.0627 (5)	
N3	0.6957 (2)	0.1904 (2)	0.98090 (13)	0.0622 (6)	
O1	0.1533 (2)	−0.19460 (12)	0.64103 (12)	0.0619 (4)	
O2	0.2245 (2)	−0.05029 (12)	0.58598 (11)	0.0553 (4)	
O3	1.1580 (3)	−0.01462 (18)	1.30023 (15)	0.0869 (6)	
O4	1.2085 (3)	0.00787 (19)	1.16010 (15)	0.0920 (7)	
O5	0.6401 (2)	0.10646 (14)	1.39061 (11)	0.0631 (4)	
H5	0.5829	0.1229	1.4306	0.095*	
O6	0.4375 (2)	0.19827 (14)	1.29741 (10)	0.0645 (5)	
O7	0.6592 (3)	0.27809 (18)	0.97449 (14)	0.0812 (6)	
O8	0.7103 (3)	0.13644 (19)	0.91429 (12)	0.0894 (7)	
O1S	0.3165 (2)	0.24418 (17)	0.61251 (13)	0.0775 (5)	
O2S	0.4987 (2)	0.16498 (16)	0.53723 (12)	0.0749 (6)	
C1	0.8417 (3)	0.08358 (16)	1.25915 (15)	0.0477 (5)	
H1	0.8824	0.0633	1.3214	0.057*	
C2	0.9446 (3)	0.06669 (17)	1.19241 (16)	0.0493 (5)	
C3	0.8917 (3)	0.09838 (17)	1.10043 (16)	0.0513 (5)	
H3	0.9632	0.0879	1.0561	0.062*	
C4	0.7316 (3)	0.14554 (17)	1.07577 (13)	0.0478 (5)	
C5	0.6150 (3)	0.16232 (16)	1.13960 (13)	0.0440 (5)	
C6	0.6781 (3)	0.13053 (16)	1.23462 (13)	0.0441 (4)	
C7	0.5720 (3)	0.14879 (17)	1.30933 (14)	0.0489 (5)	
C8	0.3398 (3)	0.20904 (16)	1.02443 (13)	0.0446 (5)	
C9	0.2480 (3)	0.29477 (18)	0.99475 (16)	0.0523 (5)	
H9	0.2679	0.3517	1.0311	0.063*	
C10	0.1267 (3)	0.29411 (17)	0.91050 (16)	0.0529 (5)	
H10	0.0614	0.3509	0.8917	0.063*	
C11	0.0978 (2)	0.21138 (16)	0.85216 (14)	0.0439 (5)	
C12	0.1923 (2)	0.12511 (15)	0.88017 (13)	0.0396 (4)	
C13	0.3113 (3)	0.12563 (16)	0.96844 (13)	0.0437 (4)	
H13	0.3724	0.0682	0.9896	0.052*	
C14	−0.0350 (3)	0.21756 (17)	0.76050 (15)	0.0514 (5)	
H14A	0.0206	0.2489	0.7132	0.062*	
H14B	−0.1345	0.2583	0.7695	0.062*	
C15	−0.1040 (3)	0.11764 (17)	0.72472 (15)	0.0480 (5)	
H15A	−0.1768	0.1243	0.6618	0.058*	
H15B	−0.1785	0.0911	0.7660	0.058*	
C16	0.0500 (2)	0.04646 (15)	0.72138 (13)	0.0393 (4)	
H16	0.1279	0.0787	0.6842	0.047*	
C17	0.1646 (3)	0.02907 (14)	0.82312 (13)	0.0402 (4)	
C18	0.0684 (3)	−0.04380 (18)	0.87898 (15)	0.0540 (5)	
H18A	0.1465	−0.0589	0.9389	0.065*	
H18B	−0.0375	−0.0124	0.8927	0.065*	
C19	0.0150 (4)	−0.13884 (19)	0.82600 (18)	0.0622 (6)	
H19A	−0.0472	−0.1808	0.8633	0.075*	
H19B	0.1209	−0.1733	0.8162	0.075*	
C20	−0.1057 (3)	−0.11764 (19)	0.73048 (18)	0.0584 (6)	
H20A	−0.2146	−0.0869	0.7409	0.070*	
H20B	−0.1377	−0.1793	0.6976	0.070*	
C21	−0.0177 (3)	−0.05074 (16)	0.66828 (14)	0.0444 (4)	
C22	−0.1505 (3)	−0.0292 (2)	0.57444 (17)	0.0612 (6)	
H22A	−0.1035	0.0220	0.5405	0.092*	
H22B	−0.2626	−0.0086	0.5883	0.092*	
H22C	−0.1677	−0.0878	0.5364	0.092*	
C23	0.1299 (3)	−0.10728 (17)	0.63367 (14)	0.0460 (5)	
C24	0.3485 (3)	−0.01273 (18)	0.81485 (15)	0.0499 (5)	
H24A	0.4068	0.0322	0.7794	0.075*	
H24B	0.3330	−0.0751	0.7829	0.075*	
H24C	0.4200	−0.0213	0.8770	0.075*	
C25	0.3532 (4)	−0.1005 (2)	0.5418 (2)	0.0760 (8)	
H25A	0.4443	−0.1289	0.5897	0.114*	
H25B	0.4062	−0.0545	0.5050	0.114*	
H25C	0.2946	−0.1517	0.5012	0.114*	
C1S	0.4227 (5)	0.3569 (3)	0.7347 (3)	0.1153 (14)	
H1S1	0.4138	0.4133	0.6939	0.173*	
H1S2	0.5131	0.3683	0.7901	0.173*	
H1S3	0.3103	0.3460	0.7531	0.173*	
C2S	0.4685 (4)	0.2728 (3)	0.6858 (2)	0.1023 (12)	
H2S1	0.5012	0.2190	0.7300	0.123*	
H2S2	0.5701	0.2878	0.6572	0.123*	
C3S	0.3494 (3)	0.19013 (19)	0.54227 (16)	0.0568 (6)	
C4S	0.1866 (4)	0.1646 (2)	0.47246 (19)	0.0685 (7)	
H4S1	0.1995	0.1859	0.4106	0.103*	
H4S2	0.0855	0.1966	0.4894	0.103*	
H4S3	0.1693	0.0946	0.4721	0.103*	

### Atomic displacement parameters (Å^2^)

**Table:**

	*U*^11^	*U*^22^	*U*^33^	*U*^12^	*U*^13^	*U*^23^
N1	0.0465 (9)	0.0698 (13)	0.0344 (8)	0.0096 (9)	0.0056 (7)	−0.0083 (8)
N2	0.0529 (11)	0.0655 (14)	0.0658 (14)	0.0085 (9)	0.0023 (10)	−0.0114 (10)
N3	0.0460 (10)	0.0995 (19)	0.0428 (10)	−0.0062 (11)	0.0133 (8)	−0.0005 (11)
O1	0.0686 (10)	0.0477 (10)	0.0715 (11)	0.0040 (8)	0.0189 (9)	−0.0004 (8)
O2	0.0591 (9)	0.0528 (9)	0.0593 (9)	0.0054 (7)	0.0249 (7)	0.0035 (7)
O3	0.0812 (13)	0.1000 (15)	0.0742 (13)	0.0356 (11)	0.0025 (10)	0.0020 (11)
O4	0.0618 (11)	0.129 (2)	0.0892 (14)	0.0323 (11)	0.0234 (11)	−0.0025 (12)
O5	0.0734 (11)	0.0769 (12)	0.0417 (8)	0.0163 (9)	0.0178 (8)	0.0080 (8)
O6	0.0606 (9)	0.0897 (13)	0.0448 (8)	0.0209 (9)	0.0144 (7)	−0.0002 (8)
O7	0.0831 (13)	0.0916 (17)	0.0666 (12)	−0.0033 (11)	0.0091 (10)	0.0213 (11)
O8	0.0761 (12)	0.154 (2)	0.0423 (9)	0.0142 (13)	0.0212 (8)	−0.0121 (11)
O1S	0.0567 (10)	0.1045 (15)	0.0693 (11)	0.0140 (10)	0.0071 (9)	−0.0255 (10)
O2S	0.0632 (10)	0.1121 (17)	0.0497 (9)	0.0202 (10)	0.0117 (8)	−0.0045 (9)
C1	0.0501 (12)	0.0453 (12)	0.0436 (11)	−0.0005 (9)	−0.0007 (9)	−0.0074 (9)
C2	0.0429 (11)	0.0503 (13)	0.0528 (13)	0.0018 (9)	0.0051 (9)	−0.0129 (9)
C3	0.0452 (11)	0.0621 (14)	0.0476 (12)	−0.0033 (10)	0.0115 (9)	−0.0154 (10)
C4	0.0440 (11)	0.0609 (14)	0.0374 (10)	−0.0035 (9)	0.0054 (8)	−0.0080 (9)
C5	0.0428 (10)	0.0505 (12)	0.0376 (10)	−0.0016 (9)	0.0058 (8)	−0.0083 (8)
C6	0.0459 (10)	0.0475 (12)	0.0377 (10)	−0.0017 (9)	0.0057 (8)	−0.0073 (9)
C7	0.0529 (12)	0.0546 (14)	0.0384 (11)	0.0010 (10)	0.0070 (9)	−0.0061 (9)
C8	0.0405 (10)	0.0557 (13)	0.0374 (10)	0.0030 (9)	0.0072 (8)	−0.0016 (9)
C9	0.0539 (12)	0.0534 (14)	0.0484 (12)	0.0042 (10)	0.0075 (10)	−0.0078 (10)
C10	0.0536 (12)	0.0484 (13)	0.0542 (13)	0.0127 (10)	0.0048 (10)	0.0010 (10)
C11	0.0396 (10)	0.0485 (12)	0.0420 (11)	0.0067 (9)	0.0043 (8)	0.0024 (9)
C12	0.0366 (9)	0.0459 (11)	0.0363 (9)	0.0033 (8)	0.0072 (7)	0.0033 (8)
C13	0.0413 (10)	0.0502 (12)	0.0387 (10)	0.0084 (9)	0.0059 (8)	0.0029 (9)
C14	0.0490 (12)	0.0506 (13)	0.0505 (12)	0.0127 (10)	−0.0005 (9)	0.0036 (10)
C15	0.0405 (10)	0.0548 (13)	0.0454 (11)	0.0089 (9)	0.0007 (9)	0.0001 (10)
C16	0.0345 (9)	0.0438 (11)	0.0392 (10)	0.0012 (8)	0.0060 (8)	0.0031 (8)
C17	0.0411 (10)	0.0419 (11)	0.0364 (10)	0.0053 (8)	0.0048 (8)	0.0016 (8)
C18	0.0690 (14)	0.0514 (13)	0.0444 (11)	0.0034 (11)	0.0177 (10)	0.0085 (10)
C19	0.0843 (17)	0.0474 (14)	0.0618 (15)	−0.0087 (12)	0.0312 (13)	0.0079 (11)
C20	0.0551 (13)	0.0555 (15)	0.0683 (16)	−0.0136 (11)	0.0211 (12)	−0.0086 (11)
C21	0.0388 (10)	0.0475 (12)	0.0456 (11)	−0.0003 (9)	0.0055 (8)	−0.0004 (9)
C22	0.0495 (13)	0.0671 (16)	0.0598 (14)	0.0015 (11)	−0.0069 (10)	−0.0117 (12)
C23	0.0441 (11)	0.0488 (13)	0.0430 (11)	−0.0010 (9)	0.0033 (9)	−0.0014 (9)
C24	0.0423 (11)	0.0553 (13)	0.0485 (12)	0.0117 (9)	0.0004 (9)	−0.0027 (10)
C25	0.0725 (17)	0.0785 (19)	0.088 (2)	0.0140 (14)	0.0429 (16)	0.0006 (14)
C1S	0.100 (3)	0.107 (3)	0.142 (3)	−0.026 (2)	0.031 (2)	−0.049 (3)
C2S	0.077 (2)	0.136 (3)	0.083 (2)	0.019 (2)	−0.0077 (17)	−0.041 (2)
C3S	0.0628 (14)	0.0618 (15)	0.0457 (12)	0.0060 (12)	0.0103 (10)	0.0077 (11)
C4S	0.0693 (16)	0.0697 (17)	0.0609 (15)	−0.0036 (12)	−0.0010 (12)	0.0105 (12)

### Geometric parameters (Å, º)

**Table:**

N1—C5	1.353 (3)	C14—H14B	0.9700
N1—C8	1.423 (3)	C15—C16	1.532 (3)
N1—H1S	0.8600	C15—H15A	0.9700
N2—O4	1.217 (3)	C15—H15B	0.9700
N2—O3	1.221 (3)	C16—C21	1.562 (3)
N2—C2	1.461 (3)	C16—C17	1.570 (3)
N3—O7	1.223 (3)	C16—H16	0.9800
N3—O8	1.230 (3)	C17—C24	1.543 (3)
N3—C4	1.472 (3)	C17—C18	1.550 (3)
O1—C23	1.202 (3)	C18—C19	1.515 (4)
O2—C23	1.338 (3)	C18—H18A	0.9700
O2—C25	1.445 (3)	C18—H18B	0.9700
O5—C7	1.316 (3)	C19—C20	1.525 (4)
O5—H5	0.8200	C19—H19A	0.9700
O6—C7	1.213 (3)	C19—H19B	0.9700
O1S—C3S	1.314 (3)	C20—C21	1.523 (3)
O1S—C2S	1.461 (4)	C20—H20A	0.9700
O2S—C3S	1.209 (3)	C20—H20B	0.9700
C1—C2	1.376 (3)	C21—C23	1.528 (3)
C1—C6	1.387 (3)	C21—C22	1.550 (3)
C1—H1	0.9300	C22—H22A	0.9600
C2—C3	1.376 (3)	C22—H22B	0.9600
C3—C4	1.365 (3)	C22—H22C	0.9600
C3—H3	0.9300	C24—H24A	0.9600
C4—C5	1.420 (3)	C24—H24B	0.9600
C5—C6	1.425 (3)	C24—H24C	0.9600
C6—C7	1.491 (3)	C25—H25A	0.9600
C8—C13	1.383 (3)	C25—H25B	0.9600
C8—C9	1.384 (3)	C25—H25C	0.9600
C9—C10	1.374 (3)	C1S—C2S	1.422 (5)
C9—H9	0.9300	C1S—H1S1	0.9600
C10—C11	1.395 (3)	C1S—H1S2	0.9600
C10—H10	0.9300	C1S—H1S3	0.9600
C11—C12	1.394 (3)	C2S—H2S1	0.9700
C11—C14	1.501 (3)	C2S—H2S2	0.9700
C12—C13	1.410 (3)	C3S—C4S	1.479 (4)
C12—C17	1.534 (3)	C4S—H4S1	0.9600
C13—H13	0.9300	C4S—H4S2	0.9600
C14—C15	1.510 (3)	C4S—H4S3	0.9600
C14—H14A	0.9700		
			
C5—N1—C8	127.69 (16)	C24—C17—C18	109.75 (18)
C5—N1—H1S	116.2	C12—C17—C16	111.36 (16)
C8—N1—H1S	116.2	C24—C17—C16	109.47 (15)
O4—N2—O3	123.7 (2)	C18—C17—C16	110.21 (17)
O4—N2—C2	118.3 (2)	C19—C18—C17	113.07 (18)
O3—N2—C2	118.0 (2)	C19—C18—H18A	109.0
O7—N3—O8	125.2 (2)	C17—C18—H18A	109.0
O7—N3—C4	118.2 (2)	C19—C18—H18B	109.0
O8—N3—C4	116.6 (3)	C17—C18—H18B	109.0
C23—O2—C25	115.90 (19)	H18A—C18—H18B	107.8
C7—O5—H5	109.5	C18—C19—C20	110.4 (2)
C3S—O1S—C2S	117.3 (2)	C18—C19—H19A	109.6
C2—C1—C6	120.7 (2)	C20—C19—H19A	109.6
C2—C1—H1	119.7	C18—C19—H19B	109.6
C6—C1—H1	119.7	C20—C19—H19B	109.6
C1—C2—C3	121.4 (2)	H19A—C19—H19B	108.1
C1—C2—N2	119.9 (2)	C19—C20—C21	112.58 (18)
C3—C2—N2	118.7 (2)	C19—C20—H20A	109.1
C4—C3—C2	118.41 (19)	C21—C20—H20A	109.1
C4—C3—H3	120.8	C19—C20—H20B	109.1
C2—C3—H3	120.8	C21—C20—H20B	109.1
C3—C4—C5	123.6 (2)	H20A—C20—H20B	107.8
C3—C4—N3	115.45 (18)	C20—C21—C23	109.39 (18)
C5—C4—N3	120.53 (19)	C20—C21—C22	109.59 (19)
N1—C5—C4	124.00 (18)	C23—C21—C22	102.13 (17)
N1—C5—C6	120.19 (17)	C20—C21—C16	111.04 (17)
C4—C5—C6	115.80 (19)	C23—C21—C16	112.97 (16)
C1—C6—C5	120.14 (18)	C22—C21—C16	111.35 (18)
C1—C6—C7	119.03 (19)	C21—C22—H22A	109.5
C5—C6—C7	120.83 (19)	C21—C22—H22B	109.5
O6—C7—O5	122.96 (19)	H22A—C22—H22B	109.5
O6—C7—C6	123.90 (19)	C21—C22—H22C	109.5
O5—C7—C6	113.13 (19)	H22A—C22—H22C	109.5
C13—C8—C9	119.86 (18)	H22B—C22—H22C	109.5
C13—C8—N1	120.96 (18)	O1—C23—O2	122.0 (2)
C9—C8—N1	119.06 (19)	O1—C23—C21	125.1 (2)
C10—C9—C8	118.7 (2)	O2—C23—C21	112.64 (19)
C10—C9—H9	120.6	C17—C24—H24A	109.5
C8—C9—H9	120.6	C17—C24—H24B	109.5
C9—C10—C11	122.41 (19)	H24A—C24—H24B	109.5
C9—C10—H10	118.8	C17—C24—H24C	109.5
C11—C10—H10	118.8	H24A—C24—H24C	109.5
C12—C11—C10	119.49 (18)	H24B—C24—H24C	109.5
C12—C11—C14	121.58 (18)	O2—C25—H25A	109.5
C10—C11—C14	118.94 (18)	O2—C25—H25B	109.5
C11—C12—C13	117.52 (18)	H25A—C25—H25B	109.5
C11—C12—C17	123.47 (16)	O2—C25—H25C	109.5
C13—C12—C17	118.90 (16)	H25A—C25—H25C	109.5
C8—C13—C12	121.94 (18)	H25B—C25—H25C	109.5
C8—C13—H13	119.0	C2S—C1S—H1S1	109.5
C12—C13—H13	119.0	C2S—C1S—H1S2	109.5
C11—C14—C15	112.35 (17)	H1S1—C1S—H1S2	109.5
C11—C14—H14A	109.1	C2S—C1S—H1S3	109.5
C15—C14—H14A	109.1	H1S1—C1S—H1S3	109.5
C11—C14—H14B	109.1	H1S2—C1S—H1S3	109.5
C15—C14—H14B	109.1	C1S—C2S—O1S	109.5 (3)
H14A—C14—H14B	107.9	C1S—C2S—H2S1	109.8
C14—C15—C16	111.05 (18)	O1S—C2S—H2S1	109.8
C14—C15—H15A	109.4	C1S—C2S—H2S2	109.8
C16—C15—H15A	109.4	O1S—C2S—H2S2	109.8
C14—C15—H15B	109.4	H2S1—C2S—H2S2	108.2
C16—C15—H15B	109.4	O2S—C3S—O1S	122.1 (2)
H15A—C15—H15B	108.0	O2S—C3S—C4S	124.9 (2)
C15—C16—C21	111.52 (17)	O1S—C3S—C4S	113.0 (2)
C15—C16—C17	110.93 (16)	C3S—C4S—H4S1	109.5
C21—C16—C17	113.55 (16)	C3S—C4S—H4S2	109.5
C15—C16—H16	106.8	H4S1—C4S—H4S2	109.5
C21—C16—H16	106.8	C3S—C4S—H4S3	109.5
C17—C16—H16	106.8	H4S1—C4S—H4S3	109.5
C12—C17—C24	108.83 (16)	H4S2—C4S—H4S3	109.5
C12—C17—C18	107.17 (15)		

### Hydrogen-bond geometry (Å, º)

**Table:**

*D*—H···*A*	*D*—H	H···*A*	*D*···*A*	*D*—H···*A*
N1—H1*S*···O6	0.86	2.02	2.654 (3)	129
O5—H5···O2*S*^i^	0.82	1.87	2.676 (3)	168
C4*S*—H4*S*3···O2	0.96	2.54	3.332 (4)	139
C15—H15*B*···O8	0.97	2.53	3.326 (4)	140

Symmetry code: (i) *x*, *y*, *z*+1.
